# Serum hepatitis B core-related antigen is a satisfactory surrogate marker of intrahepatic covalently closed circular DNA in chronic hepatitis B

**DOI:** 10.1038/s41598-017-00111-0

**Published:** 2017-03-14

**Authors:** En-Qiang Chen, Shu Feng, Meng-Lan Wang, Ling-Bo Liang, Ling-Yun Zhou, Ling-Yao Du, Li-Bo Yan, Chuan-Min Tao, Hong Tang

**Affiliations:** 1 Center of Infectious Diseases, West China Hospital, Sichuan University, Chengdu, P.R. China; 2 Division of Infectious Diseases, State Key Laboratory of Biotherapy, Sichuan University, Chengdu, P.R. China; 3 Department of Laboratory Medicine, West China Hospital, Sichuan University, Chengdu, P.R. China

## Abstract

Recently, hepatitis B core-related antigen (HBcrAg) has been suggested as an additional marker of hepatitis B virus (HBV) infection. This study aimed to investigate whether serum quantitative HBcrAg (qHBcrAg) was a satisfactory surrogate marker of intrahepatic covalently closed circular DNA (cccDNA). A total of 139 patients with liver biopsy were enrolled, consisting of 59 patients in immune tolerance (IT) phase, 52 patients in immune clearance (IC) phase, 18 patients in low-replication (LR) phase, and 10 patients in reactivation phase. All patients in IC phase have received entecavir (ETV) therapy, and 32 of them undergone a second liver biopsy at 24 months. Among those patients, qHBcrAg was strongly correlated with intrahepatic cccDNA, which is superior to that of qHBsAg and HBV DNA. And similar findings were also observed in patients in IT, IC, LR and reactivation phases. Among the 32 ETV-treated patients with a second liver biopsy in IC phase, the decline of intrahepatic cccDNA was accompanied by changes in both qHBcrAg and qHBsAg. However, as compared to qHBsAg, the change of qHBcrAg was more strongly associated with intrahepatic cccDNA-decline. In summary, serum qHBcrAg should be a satisfactory surrogate of intrahepatic HBV cccDNA in CHB patients.

## Introduction

Chronic hepatitis B (CHB) is a potentially life-threatening liver disease caused by hepatitis B virus (HBV) chronic infection. It remains a major global health issue affecting approximately 250 million people worldwide, especially in Asian countries^1^. For a long time, controlling the intrahepatic HBV covalent closed circular DNA (cccDNA) level or its transcriptional activity is critical for achieving the goals of antiviral therapy to prevent the occurrence of decompensated cirrhosis and hepatocellular carcinoma^2, 3^. So monitoring the dynamic changes of intrahepatic HBV cccDNA would be an accurate approach to evaluate the efficacy of current antiviral therapy^4, 5^.

As we know, liver biopsy is an invasive procedure, with pain and major complications occurring in 40% and 0.5% of patients, respectively^6^. In real-life clinical practice, routine liver biopsy has not been well accepted by patients, and few of them could undergo dynamic liver biopsy examinations. And this embarrassment of liver biopsy has brought great difficulties to the evaluation of intrahepatic HBV cccDNA levels in CHB patients. Thus, searching for surrogate indicators of intrahepatic HBV cccDNA has always been a research hot spot in CHB studies. Though sustained serum HBV DNA suppression and HBeAg seroconversion are reported to be associated with disease remission^2^, their efficiency in reflecting changes of intrahepatic cccDNA is poor. In recent years, quantitative HBsAg (qHBsAg) has been proven to have a better correlation with intrahepatic cccDNA than either HBV DNA or HBeAg. Besides, a new enzyme immunoassay that detects hepatitis B core-related antigen (HBcrAg) also has been reported to reflect intrahepatic cccDNA recently^7, 8^. According to reports in the literature, HBcrAg consists of HBcAg, HBeAg, and p22cr, which is a precore protein from amino acid −28 to at least amino acid 150, by coding the precore/core region^9^. However, this new serum indicator “HBcrAg” is still not widely known by clinician around the world, because there is not enough data available at present.

China is a country with the largest HBV infections in the world, and its epidemiological characteristics of CHB are different from that in other regions, especially in the United States and Europe. In present study, we will firstly investigate the distribution of serum qHBcrAg in real-life Chinese patients, assess the correlation of serum qHBcrAg with intrahepatic cccDNA, and evaluate whether serum qHBcrAg has superiority than serum qHBsAg in reflecting intrahepatic cccDNA.

## Methods

### Patients

This was a retrospective cohort study consisting of CHB patients who underwent percutaneous liver biopsy at the West China Hospital between January 2009 and December 2013. Patients were excluded if they had co-infections (HCV and HIV) or other concomitant liver diseases such as alcoholic liver disease, autoimmune liver disease, and hepatocellular carcinoma. Patients without qualified serum (enough volume and qualified storage conditions) and liver tissue samples (no obvious degradation of DNA) for qHBcrAg and intrahepatic HBV cccDNA analysis were also excluded in this study.

A total of 139 eligible patients were included and classified into different phases of CHB, consisting of 59 patients in immune tolerance (IT) phase, 52 patients in immune clearance (IC) phase, 18 patients in low-replication (LR) phase, and 10 patients in reactivation phase. The criteria were based on the 2015 Asian-Pacific Association for the Study of the Liver clinical practice guidelines on the management of hepatitis B^10^. All patients in IC phase have received entecavir (ETV) therapy, and 32 of them undergone a second liver biopsy at 24 months after antiviral therapy. This retrospective study conformed to the Ethical Guidelines of the 1975 Declaration of Helsinki. Approval of this study was also obtained from the Ethics Committee of West China Hospital of Sichuan University, and verbal informed consent was obtained from each patient in this study.

### General laboratory evaluation

Serum ALT levels were assessed according to standard procedures (Olympus AU5400, Olympus Corporation, Tokyo, Japan), and the upper limit of normal (ULN) ALT was defined as 50 IU/L for men and 38 IU/L for women. Serum HBsAg concentration was quantitatively assessed using Elecsys® HBsAg II Quant Assay (Roche Diagnostics, Penzberg, Germany), with a dynamic range of 20 to 52,000  IU/mL. If qHBsAg levels >52,000  IU/mL, samples were retested with a stepwise dilution of 1:4000. Serum hepatitis B e antigen (HBeAg) was determined by the electrochemiluminescence immunoassay (Roche Diagnostics, Indianapolis, IN, USA). Serum concentrations of HBV-DNA were determined using Cobas Taqman assay kit (Roche Diagnostics, Branchburg, NJ), with a lower limit of detection of 20 IU/mL. HBV genotypes were determined by direct S-gene sequencing.

### Quantitative intrahepatic HBV cccDNA evaluation

Besides the role of an internal reference, β-actin could also act as an effective tools to distinguish qualified tissues from unqualified ones. The positive β-actin amplification is generally believable when positive signal is acquired within 30 cycles of PCR qualification. Otherwise it should be considered as a false signal and referring to DNA degradation of host cells. In present study, β-actin amplification was applied to quantify the cellular DNA of tissue samples, and positive signal of β-actin amplification was acquired within 25 cycles in all the samples. In other words, these liver samples were all validated tissues without obvious DNA degradation and could be used for further analysis.

Intrahepatic HBV cccDNA levels were measured with the real-time PCR method^11^. The total HBV DNA was extracted from paraffin embedded liver tissue using QIAamp DNA FFPE tissue assay kit (Qiagen, Hilden, Germany). After extraction, the products were incubated with plasmid-safe ATP-dependent (PSAD) DNase (Epicentre Technologies Corp., Chicago, USA) which selectively remove linear dsDNA, linear and close-circular ssDNA, to acquire purified cccDNA. Then a rolling circle amplification (RCA) with four pairs of primers (Table 1) were conducted to increase the amplification efficiency. With such pre-treatment, the sequential RT-PCR could detected an extremely low intrahepatic cccDNA.

**Table 1:** Primers and probes used for HBV cccDNA amplification and detection.

Name	Sequence (5′ → 3′)	Nt position	Polarity
Primers for rolling circle amplification			
RCA1	AATCCTCACAATA*C*C	99–113	Sense
RCA2	ACCTATTCTCCTC*C*C	1758–1744	Anti-sense
RCA3	CCTATGGGAGTGG*G*C	510–524	Sense
RCA4	CCTTTGTCCAAGG*G*C	2689–2675	Anti-sense
RCA5	ATGCAACTTTTTC*A*C	1686–1700	Sense
RCA6	CTAGCAGAGCTTG*G*T	29–15	Anti-sense
RCA7	TAGAAGAAGAACT *C*C	2240–2254	Sense
RCA8	GGGCCCACATATT*G*T	2599–2585	Anti-sense
Primers and probes for HBV cccDNA amplification			
ccc-up	GGGGCGCACCTCTCTTTA	1523–1540	Sense
ccc-down	AGGCACAGCTTGGAGGC	1886–1870	Anti-sense
ccc-probe	FAM- TCACCTCTGCCTAATCATCTC-TAMRA	1825–1845	
Primers and probes for β-actin amplification			
β-up	ACTGTGCCCATCTACGAGG	557–576	Sense
β-down	CAGGCAGCTCGTAGCTCTT	786–803	Anti-sense
β-probe	FAM-CGGGAAATCGTGCGTGAC-TAMRA	689–706	

Note: *Indicates phosphorothioate modifications.

The cccDNA-selective primers and a probe targeting the gap region between the viral genome direct repeat regions (DR1 and DR2) were used for HBV cccDNA specifically amplification and quantification (Table 1). While the cellular DNA was quantified by determining the copy number of the cellular β-actin house keeping gene, and primers and probe for β-actin amplification were also listed in Table 1. Additionally, ten-fold serial dilutions (10^2^–10^9^ copies/mL) of a plasmid containing the entire wild-type HBV genotype C genome were used to establish standard curves for the quantitation of HBV cccDNA, and this plasmid was constructed and storage in the laboratory of our department.

In the process of detection, each sample was run in duplicate on the same plate. Liver biopsy tissues from HBV-uninfected patients were used as negative controls. The amount of HBV cccDNA was expressed as the number of copies per cell, with the estimation of 6.667 pg of DNA/cell.

### Quantitative serum HBcrAg evaluation

The quantification of HBcrAg was performed using fully automated Lumipulse chemiluminescence enzyme immunoassay (CLEIA) analyser (Fujirebio Inc., Tokyo, Japan), as described previously. Briefly, serum was incubated with pretreatment containing sodium dodecyl sulphate and then incubated with monoclonal antibodies against denatured HBcAg and HBeAg. After washing and incubated with secondary antibodies, the concentrations of HBcrAg were determined by relative chemiluminescence intensity and compared with standard curve. Because the general analytic measurement range of this assay was between 1,000 U/ml (3 log10 U/ml) and 10,000,000 U/ml (7 log10 U/ml), serial dilutions of the serum sample is needed when serum qHBcrAg level above the detection limit of the assay.

### Statistical analyses

Data were expressed as mean and SD for continuous variables and as counts and percentages for categorical variables. The differences between continuous variables were analyzed using the Student’s t test or Mann–Whitney test, as appropriate; and the comparison of continuous variables before and after antiviral therapy was analyzed using paired samples t test, considering a *P* value less than 0.05 as statistically significant. The correlation between two continuous variables was analyzed using Spearman’s bivariate correlation, and the correlation is significant at the 0.01 level (2-tailed). All statistical analyses were done with SPSS version 18.0 (SPSS, Chicago, IL), and all figures were drawn using GraphPad Prism 6 (GraphPad Software Inc., California, USA).

## Results

### Patient characteristics

Among the 139 eligible patients, there were more males (69.06%) than females, HBeAg-positive patients (79.86%) than HBeAg-negative patients, and HBV Genotype B (63.31%) than C. The mean levels of intrahepatic HBV cccDNA was 7.33 ± 1.03 log10 copies/10^6^ cell; and mean levels of serum HBV DNA and HBsAg was 6.99 ± 1.85 log10 IU/mL and 4.15 ± 0.86 log10 IU/mL, respectively. The detailed characteristics of patients in total and in different phases of CHB were presented in Table 2

**Table 2:** Demographic and clinical characteristics of patients in this study.

Variables	Total (n = 139)	Immune-tolerant phase (n = 59)	Immune-clearance phase (n = 52)	Low-replicative phase (n = 18)	Reactivation phase (n = 10)
Age (yr)	32.09 ± 7.78	31.58 ± 7.15^#^	29.23 ± 7.19^*^	38.11 ± 5.27^*#^	39.20 ± 8.72
Gender (M/F)	96/43	40/19	38/14	12/6	6/4
Smoke (Y/N)	17/122	5/54	7/45	3/15	2/8
Alcohol intake (Y/N)	5/134	3/56	1/51	1/17	0/10
ALT (IU/L)	66.09 ± 74.67	24.93 ± 10.55^*^	134.77 ± 84.93^*#^	26.00 ± 11.28^#^	23.90 ± 10.37
HBeAg (+/−)	111/28	59/0	52/0	0/18	0/10
HBV Genotype (B/C)	88/51	39/20	34/18	8/10	7/3
HBV DNA (log10 IU/mL)	6.99 ± 1.85	7.70 ± 0.89^*#^	7.88 ± 0.86	3.20 ± 0.47^*^	4.98 ± 1.21^#^
Intrahepatic cccDNA (log10 copies/10^6^ cell)	7.33 ± 1.03	7.57 ± 0.63^*#^	7.98 ± 0.41^*^	5.62 ± 0.58^#^	5.68 ± 0.52
Serum HBsAg (log10 IU/mL)	4.15 ± 0.86	4.48 ± 0.48^*#^	4.35 ± 0.81	3.06 ± 0.64*	3.14 ± 0.95#

Note: The symbols represent significant difference between two groups with *P* ≤ 0.05.

.

### Serum HBcrAg distribution in CHB patients

**Figure 1:**
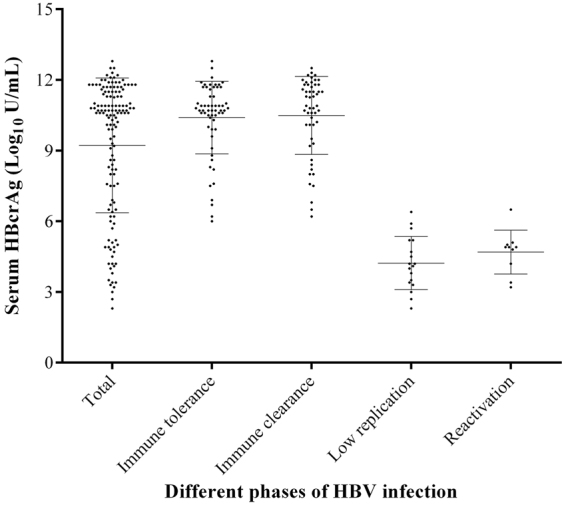
The distribution of serum qHBcrAg in different phases of CHB.

Among those 139 CHB patients, the serum qHBcrAg levels varied significantly and were widely distributed among different phases of HBV infection. As shown in Fig. 1, the level of qHBcrAg was ranged from 2.30 to 12.80 log10 U/mL, with a mean level of 9.23 ± 2.86 log10 U/mL. And the mean levels of qHBcrAg were 10.40 ± 1.54 log10 U/mL for patients in IT phase (median 10.7 log10 U/mL, range 6–12.8 log10 U/mL), 10.49 ± 1.66 log10 U/mL for patients in IC phase (median 10.9 log10 U/mL, range 6.2–12.5 log10 U/mL), 4.23 ± 1.13 log10 U/mL for patients in LR phase (median 4.15 log10 U/mL, range 2.3–6.4 log10 U/mL), and 4.69 ± 0.93 log10 U/mL for patients in reactivation phase (median 4.90 log10 U/mL, range 3.2–6.5 log10 U/mL). There was no significant difference of qHBcrAg distribution between IT phase and IC phase (*P* = 0.755), LR phase and reactivation phase (*P* = 0.439), but significant difference was observed between IT phase and LR phase (*P* < 0.001), IT phase and reactivation phase (*P* < 0.001), IC phase and LR phase (*P* < 0.001), IC phase and reactivation phase (*P* < 0.001).

**Figure 2:**
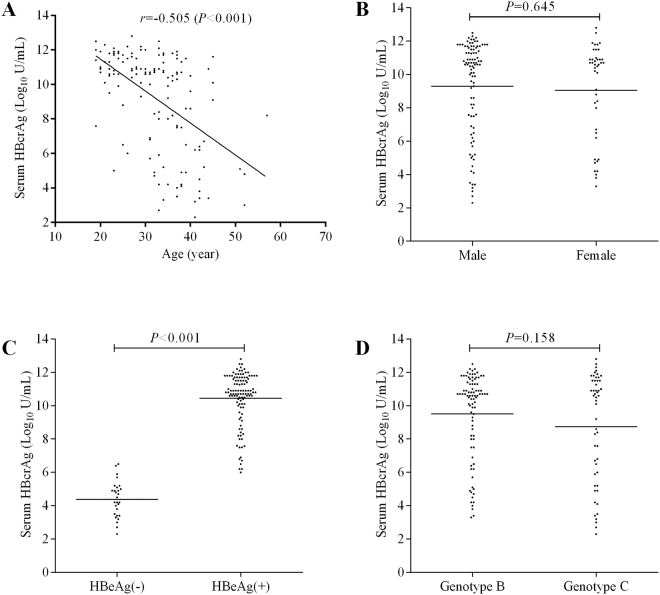
The correlation of serum qHBcrAg with age (**A**), and distribution of serum qHBcrAg distinguished by gender (**B**), HBeAg statue (**C**) and HBV genotype (**D**).

Among those 139 patients, the distribution of serum qHBcrAg was statistically negatively correlated with age (*r* = −0.505, *P* < 0.001) (Fig. 2A), but not statistically different between males and females (9.30 ± 2.86 log10 U/mL *vs.*9.06 ± 2.88 log10 U/mL, *P* = 0.645) (Fig. 2B). The serum level of qHBcrAg was significantly higher in HBeAg-positive patients than in HBeAg-negative patients (10.45 ± 1.59 log10 U/mL *vs.* 4.39 ± 1.07 log10 U/mL, *P* < 0.001) (Fig. 2C), but similar between genotype B and C HBV infected patients (9.51 ± 2.58 log10 U/mL *vs.* 8.75 ± 3.26 log10 U/mL, *P* = 0.132) (Fig. 2D).

### Correlations of qHBcrAg with intrahepatic cccDNA and other clinical parameters

**Figure 3:**
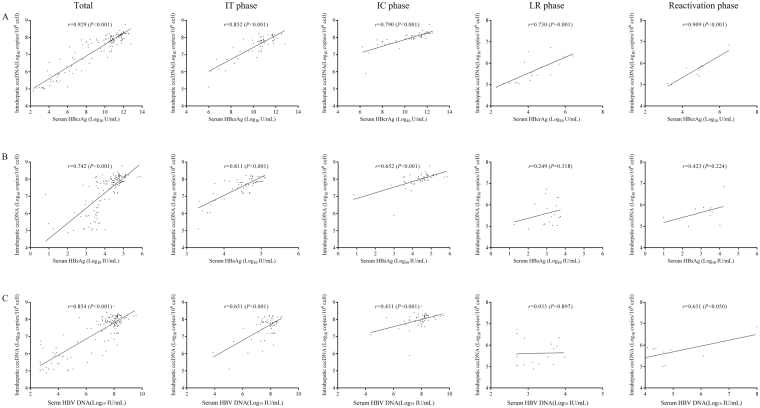
The correlations of serum qHBcrAg (**A**), qHBsAg (**B**) and HBV DNA (**C**) with intrahepatic cccDNA among different phases of CHB.

**Figure 4:**
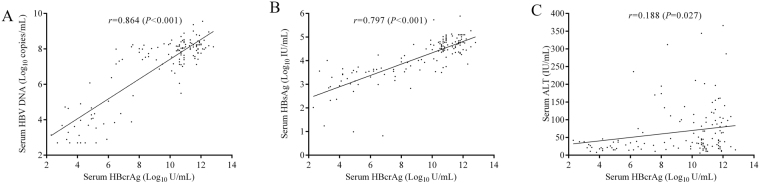
The correlations of serum qHBcrAg with serum HBV DNA (**A**), qHBsAg (**B**) and ALT (**C**) variables.

Among those 139 CHB patients, the serum qHBcrAg level was positively associated with intrahepatic cccDNA levels (*r* = 0.929, *P* < 0.001). The correlation of serum qHBcrAg level and intrahepatic cccDNA level was also statistically significant in all phases (*r* = 0.852, *P* <  0.001 for IT phase; *r* = 0.790, *P* <  0.001 for IC phase; *r* = 0.730, *P* <  0.001 for LR phase; and *r* = 0.909, *P* <  0.001 for reactivation phase) (Fig. 3A). Additionally, the serum qHBcrAg level was also positively associated with serum HBV DNA (*r* = 0.864, *P* < 0.001) and qHBsAg (*r* = 0.797, *P* < 0.001), respectively (Fig. 4A and B). However, no significant correlation was observed between the qHBcrAg level and ALT level (Fig. 4C).

Among those 139 CHB patients, serum qHBsAg (*r* = 0.742, *P* < 0.001) and HBV DNA (*r* = 0.854, *P* < 0.001) levels were also positively associated with intrahepatic cccDNA level. However, their correlations were relatively weaker than that of qHBcrAg (*r* = 0.929, *P* < 0.001). Among patients in different phases, the correlations of qHBsAg with intrahepatic cccDNA were significant in the IT (*r* = 0.811, *P* < 0.001) and IC (*r* = 0.652, *P* < 0.001) phases, but not in the LR (*r* = 0.249, *P* = 0.318) and reactivation (*r* = 0.423, *P* = 0.224) phases (Fig. 3B). Similar correlations were also observed between serum HBV DNA and intrahepatic cccDNA in the IT (*r* = 0.651, *P* < 0.001), IC (*r* = 0.431, *P* = 0.001), LR (*r* = 0.033, *P* = 0.897) and reactivation (*r* = 0.631, *P* = 0.050) phases (Fig. 3C). These findings indicated that serum qHBcrAg level was superior to either qHBsAg and HBV DNA in reflecting the intrahepatic cccDNA level in all phases of CHB.

### Dynamic changes of qHBcrAg and intrahepatic cccDNA levels during treatments

**Figure 5:**
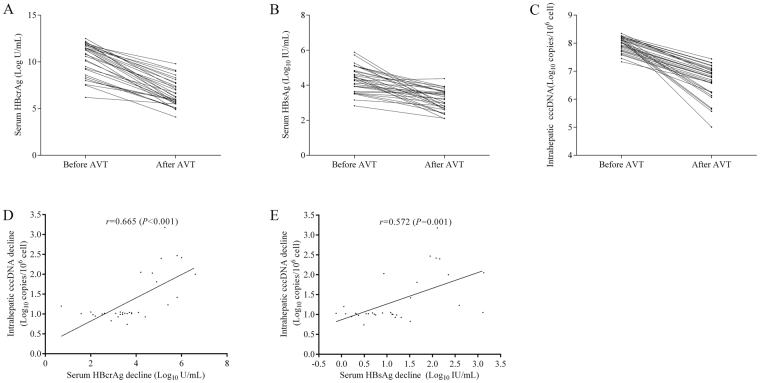
Dynamic changes of serum qHBcrAg (**A**), qHBsAg (**B**) and intrahepatic cccDNA (**C**) before and after antiviral therapy; and the correlations of dynamic changes of serum qHBcrAg (**D**) and qHBsAg (**E**) with intrahepatic cccDNA declines.

The detailed information of ETV-treated patients with dynamic liver biopsy in the IC phase were presented in Table 3. During the 24 months of ETV treatment, the reductions of serum qHBcrAg and qHBsAg were accompanied by changes in intrahepatic cccDNA. As shown in Fig. 5, the mean levels of qHBcrAg were decreased from 10.35 ± 1.73 log10 U/mL to 6.58 ± 1.39 log10 U/mL, with a mean reduction of 3.77 ± 1.38 log10 U/mL; the mean levels of qHBsAg were decreased from 4.34 ± 0.73 log10 IU/mL to 3.16 ± 0.59 log10 IU/mL, with a mean reduction of 1.19 ± 0.88 log10 IU/mL; and the mean levels of intrahepatic cccDNA were also decreased from 7.98 ± 0.26 log10 copies/10^6^ cell to 6.64 ± 0.57 log10 copies/10^6^ cell, with a mean reduction of 1.34 ± 0.61 log10 copies/10^6^ cell.

**Table 3:** The detailed information of patients receiving entecavir treatment in IC phase of CHB.

No.	Age (yr)	Sex	ALT (IU/L)	GT	HAI	Ishak	Before antiviral therapy				After antiviral therapy			
							HBcrAg (lg U/mL)	HBsAg (lg IU/mL)	HBVDNA (lg IU/mL)	HBV cccDNA (lg copies/10^6^ cell)	HBcrAg (lg U/mL)	HBsAg (lg IU/mL)	HBVDNA (lg IU/mL)	HBV cccDNA (lg copies/10^6^ cell)
1	42	M	236	B	6	1	6.20	6.26	6.36	7.46	5.50	3.59	1.69	6.26
2	22	M	107	C	1	0	11.80	7.30	8.23	8.23	8.60	3.92	2.95	7.30
3	36	M	134	B	5	0	8.20	6.59	7.99	7.61	5.10	2.99	<1.30	6.59
4	22	M	137	B	9	4	9.50	6.60	7.14	7.34	5.90	3.44	1.67	6.60
5	37	F	98	B	5	0	11.50	6.23	8.11	8.04	6.60	2.69	<1.30	6.23
6	20	M	92	B	3	1	11.90	6.91	8.23	7.94	8.10	4.38	<1.30	6.91
7	33	M	174	B	5	1	8.00	6.78	8.01	7.73	5.80	3.89	1.75	6.78
8	35	F	195	B	3	1	8.00	6.78	7.81	7.76	5.90	3.58	<1.30	6.78
9	19	M	119	C	5	0	11.40	5.65	8.31	8.05	6.30	2.35	2.49	5.65
10	33	F	106	B	10	2	10.90	5.68	8.89	8.10	4.90	2.70	2.08	5.68
11	35	M	95	C	3	1	9.20	6.79	8.09	7.81	5.70	3.52	1.97	6.79
12	33	F	312	C	5	3	8.40	6.90	7.53	7.89	5.10	3.18	1.51	6.90
13	34	M	202	B	6	0	10.80	6.90	8.03	7.91	7.00	3.34	<1.30	6.90
14	19	F	366	B	5	1	12.00	6.93	8.28	8.35	6.20	2.95	1.69	6.93
15	24	F	155	C	4	0	11.50	6.96	9.01	8.00	7.40	3.66	<1.30	6.96
16	19	F	109	B	5	0	12.50	7.02	8.96	8.25	7.10	2.68	2.37	7.02
17	19	M	170	C	12	5	7.58	6.57	8.23	7.58	6.00	2.83	1.58	6.57
18	23	M	344	B	7	2	10.60	7.05	8.23	8.10	7.30	2.42	<1.30	7.05
19	21	M	149	C	6	0	11.30	5.57	8.05	8.04	5.50	2.34	<1.30	5.57
20	28	M	197	B	4	1	11.50	7.08	8.23	8.12	7.80	3.94	2.33	7.08
21	23	M	161	B	1	0	11.80	7.11	8.23	8.16	9.80	2.78	2.82	7.11
22	40	M	111	C	6	3	8.60	6.64	7.74	7.65	5.00	2.11	<1.30	6.64
23	29	M	100	B	4	1	9.30	6.69	6.86	7.71	6.70	3.46	1.47	6.69
24	26	M	129	C	2	1	11.50	7.19	9.37	8.19	9.00	3.67	7.53	7.19
25	25	M	160	B	1	0	11.70	7.21	8.23	8.20	8.30	3.63	1.88	7.21
26	23	M	140	C	4	1	12.10	7.31	9.56	8.24	7.70	3.79	2.17	7.31
27	30	M	104	B	1	0	12.00	7.44	8.23	8.27	9.10	3.59	3.16	7.44
28	38	M	434	B	4	2	7.50	6.83	8.12	7.86	4.10	3.24	<1.30	6.83
29	21	M	115	B	2	0	10.10	6.07	8.50	8.12	5.90	2.60	<1.30	6.07
30	36	F	211	C	1	1	10.21	6.13	7.16	8.16	5.50	2.61	<1.30	6.13
31	34	M	121	B	2	1	11.26	5.01	8.18	8.19	6.00	3.02	<1.30	5.01
32	26	M	286	B	3	1	12.20	6.24	8.49	8.24	5.60	2.10	<1.30	6.24

Among these 32 ETV-treated patients in the IC phase, either before or after 24-month antiviral therapy, the correlation of qHBcrAg with intrahepatic cccDNA was higher than that of qHBsAg with intrahepatic cccDNA (*r* = 0.830 *vs. r* = 0.684 before treatment; r = 0.578 *vs.* r = 0.536 after treatment). Importantly, the dynamic changes of qHBcrAg (*r* = 0.665, *P* < 0.001) were more strongly associated with intrahepatic cccDNA-decline as compared to that of qHBsAg (*r* = 0.572, *P* = 0.001) (Fig. 5D and E).

## Discussion

It is well known that the HBV genome exists in the nuclei of infected hepatocytes as a 3.2 kb double-stranded episomal DNA species called cccDNA. The cccDNA is a key component in the HBV life cycle, since it is the template for all viral genomic and subgenomic transcripts, and its level is well correlated with the proliferative potential of HBV^12^. So monitoring the dynamic changes of intrahepatic cccDNA levels is important for accurately evaluating the effectiveness of current antiviral therapy and the risks of viral rebound resulting from discontinuation of nucleos(t)ide analogs (NAs) in CHB patients^4, 13^. However, it is regrettable that the intrahepatic cccDNA is not routinely monitored in patients in real-life clinical practice due to the inconvenience of percutaneous liver biopsy and complexity of cccDNA examination. So serum markers reflecting the intrahepatic cccDNA level may be considered as a useful surrogate indicator.

Over the past decades, serum level of HBV DNA has been reported to correlate well with intrahepatic cccDNA levels in the natural course but not under nucleos(t)ide analogues therapy, because intrahepatic cccDNA decline did not parallel the rapid decrease of serum HBV DNA during a relative short duration of NAs therapy. Recently, HBcrAg has been suggested as an additional marker of HBV infection and reported to be correlated with intrahepatic cccDNA^7, 14^. In this study, we firstly investigated the correlation of serum qHBcrAg with intrahepatic cccDNA in the different phases of CHB in Chinese mainland. It was to mention that the criteria of the four phases of CHB mentioned in this study were based on the 2015 APASL clinical practice guideline of CHB^10^, which had some difference with previous natural history definition of CHB^15^. As we expected, serum qHBcrAg not only has a significant positive correlation with intrahepatic cccDNA in nature course of CHB, but also this correlation is superior to that of serum qHBsAg with intrahepatic cccDNA. In addition, we also found that antiviral therapy could successfully reduce serum HBcrAg concentration, as well as serum HBV DNA and intrahepatic cccDNA levels, and this finding was supported by recent two similar studies^16, 17^.

Importantly, the reductions of serum HBcrAg were also significantly positive correlated with the declines of intrahepatic cccDNA during treatment. Thus, it is easy to see that serum HBcrAg could be regarded as a satisfactory surrogate indicator of intrahepatic cccDNA and a better predictor of the long-term prognosis of CHB at present. As we known, the production of HBcrAg depends on the transcription of mRNA from cccDNA, so the reduction of HBcrAg is slower than that of serum HBV DNA in patients under nucleos(t)ide analogues therapy, and this may also explain why serum HBcrAg presents a significant positive correlation with intrahepatic cccDNA before and after ETV therapy.

In this study, the mean levels of serum qHBcrAg were higher than that reported in the literature^18^. This phenomenon may be related to the high percentage of positive HBeAg in this study, because HBeAg exists in serum at much higher concentrations than HBcAg and HBcrAg concentrations. In fact, the low levels of serum qHBcrAg in HBeAg-negative patients or in the low replication/Reactivation phases of CHB in this study, also had proved this explanation.

Considering that HBV genotype may affect the level of viral replication and host immune responses, we also analyze the distribution of serum HBcrAg concentration between genotype B and C patients, but no significant difference is observed. To our knowledge, HBcrAg is a precore protein which is encoded by precor/core regions of HBV genome and unaffected by the promoters located in S region, so it is easy to understand the similar distribution of HBcrAg between different HBV genotypes.

In past decades, serum qHBsAg had been reported to be useful in reflecting intrahepatic cccDNA levels. However, as compared to serum qHBcrAg in present study, its correlation with intrahepatic cccDNA was relative weaker, not only in nature history of CHB but also during antiviral treatment. In fact, we need to know that majority of patients with HBsAg loss/seroconversion still could detect intrahepatic HBV DNA and cccDNA. For example, Prof. Kumada H *et al.* recently have detected positive HBcrAg in 6 out of 13 HBsAg seroclearance patients, and the serum level of HBcrAg is still correlated with intrahepatic cccDNA levels^7^. So, according to current available evidences, serum qHBcrAg should be more suitable than qHBsAg as an alternative indicator of intrahepatic cccDNA levels.

In this study, a negative correlation of qHBcrAg with age is also observed. Thus, an accurate comparison of serum qHBcrAg between groups should not ignore the possible interference of age and other related characteristics. Although our findings are exciting and believable, limitations are also inevitably existed. The low numbers of patients with unequal distribution in different phases of CHB should be the greatest weakness of this study. Thus, large sample (including both HBeAg positive and HBeAg-negative patients) and multi-center prospective studies are urgently needed to verify present findings. Additionally, the correlation of serum qHBcrAg and intrahepatic cccDNA dynamic declines also should be verified in peginterferon alfa-treated patients.

In conclusion, serum qHBcrAg is well correlated with intrahepatic cccDNA level in CHB, and qHBcrAg is a good candidate to be a satisfactory surrogate marker. Thus the measurement of serum qHBcrAg may be clinically useful for monitoring the viral status of intrahepatic HBV and predicting the long-term prognosis of CHB patients.
